# Higher body mass index may induce asthma among adolescents with pre-asthmatic symptoms: a prospective cohort study

**DOI:** 10.1186/1471-2458-11-542

**Published:** 2011-07-08

**Authors:** Wen-Chao Ho, Yu-Sheng Lin, James L Caffrey, Meng-Hung Lin, Hui-Tsung Hsu, Leann Myers, Pau-Chung Chen, Ruey-Shiung Lin

**Affiliations:** 1 Department of Public Health, China Medical University, Taichung City, Taiwan; 2 Institute of Environmental Health, China Medical University, Taichung City, Taiwan; 3 Department of Environmental and Occupational Health, School of Public Health, University of North Texas Health Science Centre, Fort Worth, Texas, USA; 4 Department of Integrative Physiology and Cardiovascular Research Institute, University of North Texas Health Science Centre, Fort Worth, Texas, USA; 5 Department of Health Risk Management, China Medical University, Taichung City, Taiwan; 6 Department of Biostatistics and Bioinformatics, Tulane University, New Orleans, Louisiana, USA; 7 Institute of Occupational Medicine and Industrial Hygiene and Department of Public Health, National Taiwan University College of Public Health, Taipei City, Taiwan; 8 Department of Environmental and Occupational Medicine, National Taiwan University Hospital and National Taiwan University College of Medicine, Taipei City, Taiwan; 9 Institute of Epidemiology and Preventive Medicine, National Taiwan University, Taipei City, Taiwan

## Abstract

**Background:**

Limited studies have prospectively examined the role of body mass index (BMI) as a major risk factor for asthma during adolescence. This study investigates whether BMI is associated with increased risk of developing physician-diagnosed asthma during 12-month follow-up among adolescents with undiagnosed asthma-like symptoms at baseline.

**Methods:**

A total of 4,052 adolescents with undiagnosed asthma-like symptoms at baseline were re-examined after a 12-month follow-up. Asthma cases were considered confirmed only after diagnosis by a physician based on the New England core and International Study of Asthma and Allergies in Childhood (ISAAC) criteria video questionnaires, and accompanying pulmonary function tests. Logistic regression analyses were used to evaluate the relationship of BMI and the risk of acquiring asthma.

**Results:**

The results indicated that girls with higher BMI were at an increased risk of developing asthma during the 12-month follow-up. The odds ratios for girls developing physician-diagnosed asthma were 1.75 (95% CI = 1.18-2.61) and 1.12 (95% CI = 0.76-1.67), respectively, for overweight and obesity as compared to the normal weight reference group after adjustment for other covariates. A similar relationship was not observed for overweight and obese boys who were also significantly more active than their female counterparts.

**Conclusions:**

Increased BMI exaggerates the risk of acquiring asthma in symptomatic adolescent females but not in adolescent males. Thus, gender is an important modifier of BMI-related asthma risk. Additional research will be required to determine whether the increased asthma risk results from genetic, physiological or behavioural differences.

## Background

Asthma in children has increased consistently for decades in industrialized and westernized countries [1]. The cause of this alarming trend remains unclear. Though asthma has been associated with selected genetic polymorphisms [2], environmental air quality has been a prime suspect [3]. Despite this logic, childhood asthma in Taiwan increased fifteen fold from 1.3% to 19.0% during the thirty year period, 1974 - 2003 when air pollution patterns remained consistent [3,4]. The prevalence of childhood obesity appears to be following a similar upward trend; doubling or more since the early nineties [5]. The prevalence of obesity in Taiwan increased more than 4% alone in the two year interval 1999 and 2001 [6].

There appears to be a strong link between obesity and asthma in children and adolescents [7,8]. US adolescents with a body mass index (BMI) at the 85^th ^percentile or greater were at the highest risk for developing asthma [8]. Other cross-sectional studies were less consistent [8-10]. Conflicting gender-specific associations between obesity and asthma have been reported for both girls only [9] and boys only [10]. Thus, it remains unclear whether the interaction between gender and BMI plays a role in asthma risk [11]. Other risk factors such as allergens and environmental tobacco smoking may obscure true causal relationships between BMI and asthma [12]. A review of prospective studies indicates that a greater incidence of asthma in overweight and/or obese children and adolescents especially when obesity antedates the asthma [11]. Furthermore, asthma morbidity is highest among the young, especially before or during puberty [13]. Since obesity and asthma have become major public health concern in Taiwan and the west [6], its clearly important to examine the temporal relation between body mass and asthma risk [14], especially during the formative adolescence period of rapid growth and gender expression. We hypothesize that obesity aggravates the risk of asthma. This prospective cohort study thus hopes to establish a potential relationship between BMI and physician-diagnosed asthma during 12-month follow-up among 4,052 adolescents aged 13-15 years old with undiagnosed asthmatic symptoms at study entry.

## Methods

### Study design

The present study is part of a large cohort study (n = 1,139,452) on respiratory health of junior high school students conducted by the Taiwan Environmental Protection Agency (TEPA) and National Taiwan University (NTU) from 1995 to 1996. This study has been reviewed and approved by the TEPA and the Institutional Review Board at NTU with written consent of patients and Ethics Committee approval. To achieve the quality assurance and quality control (QA/QC) in the survey, six work groups were formed including QA/QC, clinical medicine, education and training, statistics, computer science and epidemiology. Top experts in the field in Taiwan were responsible for the development of standardized questionnaires, operating and training procedures and a computerized program to identify screening errors. They meet weekly to address problems and access QA/QC.

Details of the project including the quality control program have been reported elsewhere [3]. This study selected a 20% random sample with pulmonary function tests (PFTs) from the Taipei area. Adolescents with pre-asthmatic symptoms at baseline were followed for one year to test the hypothesis that elevated BMI increases their risk of subsequently developing asthma. In brief, a total of 4,936 individuals with undiagnosed asthma-like symptoms based on the International Study of Asthma and Allergies in Childhood (ISAAC) criteria were selected by non-physician staff from a cohort of 9,546 adolescents aged 13-15 years in the Taipei area. Asthmatic symptoms, based on the ISAAC criteria, were defined as an affirmative answer to any of the four following questions: 1) Have you ever experienced wheezing or whistling in the chest at any time in the past?; 2) Has your sleep been disturbed due to wheezing?; 3) Has your chest sounded wheezy during or after exercise?, and 4) Have you had a dry cough at night?

PFTs were performed by Sensor Medics 2130 PC-based spirometer that meets 1977 American Thoracic Society (ATS) and 1987 ATS snowbird standards. PEFs included the measurement of the forced expiratory volume in one second (FEV_1_), forced vital capacity (FVC) and their ratio (FEV_1_/FVC). This study excluded 160 malnourished adolescents (BMI < 16.0 kg/m^2^) because of evidence that very low BMI is associated with impaired lung function arising from unique etiologic causes [7,15]. Individual subjects who experienced substantive changes in BMI (> 3 standard deviations, n = 49) and those missing data on key analytical variables (n = 175) or follow up PETs (n = 501) were also excluded to strengthen the normality and representativeness of study population [16]. A total of 4,052 adolescents were thus included in the analysis. There were no significant differences in demographic or asthma risk variables between cases that were included or excluded in the current analysis.

### Physician-diagnosed asthma

Physician-diagnosed asthma was based on uniform criteria that included the New England core questionnaire, the ISAAC video questionnaire [3,17], based on both parent and student answers guided by adolescent respiratory system technical consultants, and ATS-PFT standards [18]. Percent predicted PFT values were computed for each individual adjusted for age, gender, and height [19]. The reversibility criteria used in the present study were determined as FEV_1_/FVC < 70 along with the percent change in FEV_1 _[20].

### Other Risk factors for asthma

Height and weight were measured by the project staff at enrolment and 12 months later. A standardized procedure was followed to determine body height and weight including removing shoes. BMI was calculated and classified into four categories: under-weight, normal, overweight and obese, using age- and gender-specific thresholds reported previously for children and adolescents based on a nationwide study in Taiwan [21]. Information regarding other risk asthma factors was obtained during the baseline household interview using standardized questions. These inquiries included exercise frequency (seldom, sometimes, regularly), parental asthma (yes if father or mother responded affirmatively), parental education (≤ 9 yrs, 10 - 12 yrs, ≥ 13 yrs), breastfeeding (no, ≤ 1 month, > 1 month), and dichotomous variables (yes or no) for air-conditioning, cigarette smoking, environmental tobacco smoke (ETS) exposure, pet(s), and fungus/mould found in home [3,22].

### Statistical analysis

The distributions of demographic characteristics by gender were initially examined using Pearson's Chi-square test and non-parametric Wilcoxon rank sum tests (if the distributions were skewed) for categorical and continuous variables, respectively. A generalized Mantel-Haenszel Chi-square test was used to examine the relationships between BMI and these covariates at baseline. To investigate associations between BMI and asthma risk further, adjusted odds ratios (ORs) and 95% CIs were calculated using logistic regression. Exercise frequency, parents with asthma, parental education, breastfeeding, pet(s) ownership, household fungus/mould, air-conditioning usage, smoking and environmental tobacco smoke exposure were considered as potential covariates for which the ORs were adjusted. Models were also fit that included the change in BMI during 12-month follow-up as an additional covariate. The models were assessed for goodness of fit using the Hosmer and Lemeshow test. The likelihood-ratio test was also used to select a parsimonious model to characterize asthma risk. Statistical analyses were performed with the SAS 9.13 (SAS Inc., Cary, NC). The statistical significance level was set at 0.05.

## Results

Table 1 shows the proportion of adolescents with physician-diagnosed asthma at the follow-up exam by baseline demographic characteristics. In general, the occurrence was higher among girls (14.1%) than boys (10.9%) (*p *= 0.002, Chi-square test) and varied by demographic characteristics. For instance, BMI was not associated with the occurrence asthma in boys (*p *= 0.51), whereas there was a significant positive trend in girls (*p *= 0.04). Of other factors, the risk of acquiring physician-diagnosed asthma was greater among all adolescents exposed to household mould/fungal and those with a parental history of asthma. In addition, asthma was more prevalent in adolescents who were not breastfed or whose parents were better education but not in comparable female subjects. None of the other covariates (air-conditioning usage, cigarette smoking, exposure to environmental tobacco smoking, and pet ownership) attained significance (*p *= 0.05).

**Table 1:** Baseline demographic characteristics of study subjects and occurrence of physician-diagnosed asthma during the follow-up by gender

Variables	Boys		Girls	
	
	Observation, No. (%)	% with asthma	Observation, No. (%)	% with asthma
Overall	2206	10.9	1846	14.1
BMI ‡				
Underweight	307 (13.9)	8.5	204 (11.1)	11.3
Normal	1327 (60.2)	11.3	1201 (65.1)	13.5
Overweight	276 (12.5)	11.2	251 (13.6)	14.3
Obese	296 (13.4)	11.5	190 (10.3)	20.5
Breastfeeding †				
No	1284 (58.2)	12.5	1031 (55.9)	14.0
≤ 1 month	382 (17.3)	10.2	312 (16.9)	16.0
> 1 month	540 (24.5)	7.59	503 (27.3)	13.1
Exercise frequency				
Seldom	193 (8.75)	11.4	344 (18.6)	15.1
Sometimes	848 (38.4)	10.6	1081 (58.6)	13.5
Regularly	1165 (52.8)	11.1	421 (22.8)	14.7
Air conditioning				
No	230 (10.4)	10.9	213 (11.5)	17.4
Yes	1976 (89.6)	10.9	1633 (88.5)	13.7
Cigarette smoking				
No	2007 (91.0)	11.1	1800 (97.5)	13.9
Yes	199 (9.0)	9.54	46 (2.5)	19.6
Exposure to ETS				
No	956 (43.3)	11.0	777 (42.1)	15.7
Yes	1250 (56.7)	10.9	1069 (57.9)	12.9
Mould/Fungal at home *				
No	1588 (72.0)	10.1	1267 (68.6)	12.8
Yes	618 (28.0)	13.1	579 (31.4)	16.9
Parental history of asthma *				
No	2136 (96.8)	13.5	1754 (95.0)	13.5
Yes	70 (3.2)	27.1	92 (5.0)	25.0
Parent education (%) †				
≤ 6 yrs	745 (33.8)	7.92	624 (33.8)	13.5
7 - 12 yrs	769 (34.9)	12.0	681 (36.9)	12.6
≥ 13 yrs	692 (31.4)	13.0	541 (29.3)	16.6
Pet				
No	1623 (73.6)	10.8	1312 (71.1)	14.0
Yes	583 (26.4)	11.1	534 (28.9)	14.2

BMI, body mass index; ETS, environmental tobacco smoking.

* Significantly (*p *< 0.05) associated with asthma risk in both boys and girls.

† Significantly (*p *< 0.05) associated with asthma risk in boys only.

‡ Significantly (*p *< 0.05) associated with asthma risk in girls only.

Of note, significant differences between genders were found for a number of demographic and lifestyle variables, including BMI, exercise frequency, cigarette smoking, mould/fungal exposure at home, and prevalence of parents with asthma (*p *< 0.05) (Table 1). As expected, male adolescents generally had 20-25% larger lung capacity than their female counterparts. Small but statistically significant differences in the FEV_1_, FVC, and FEV_1_/FVC ratio were observed between the baseline and follow-up exams for both genders (Table 2). Furthermore, both boys and girls diagnosed with asthma had lower than predicted pulmonary function tests at follow up (Table 2).

**Table 2:** Spirometric measurements at baseline and follow-up exam*

Characteristics	Baseline						Follow-up											
	
	Boys			Girls			Boys						Girls					
							
							Asthma			Non-asthma			Asthma			Non-asthma		
FEV_1 _(L)	3.30	±	0.58	2.66	±	0.43	3.41	±	0.53	3.46	±	0.54	2.64	±	0.40	2.64	±	0.39
FEV_1 _(% of predicted)	101.5	±	12.9	97.8	±	13.8	99.5	±	12.8	99.6	±	11.9	94.8	±	11.3	95.2	±	12.1
FVC (L)	3.64	±	0.64	2.88	±	0.45	3.73	±	0.62	3.74	±	0.60	2.80	±	0.42	2.81	±	0.41
FVC (% of predicted)	99.1	±	12.5	99.2	±	13.2	96.5	±	12.2	95.9	±	11.3	94.5	±	11.2	95.2	±	11.6
FEV_1_/FVC (%)	91.1	±	6.46	92.5	±	6.78	91.9	±	5.78	92.7	±	5.63	94.2	±	5.40	94.2	±	5.36

FEV_1_, forced expiratory volume in one second; FVC, forced vital capacity.

* Change in spirometric measurements between baseline and follow-up were statistically significant (p < 0.05).

**Figure 1:**
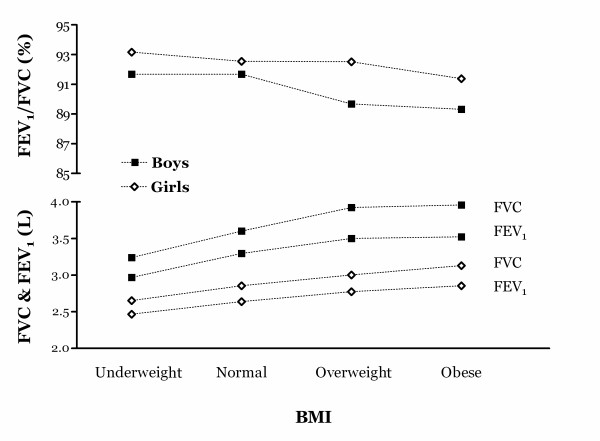
**Baseline spirometric lung functions stratified by sex and body mass index (BMI)**. All of three spirometric lung functions were significantly different across BMI categories (p < 0.05). FEV_1_, forced expiratory volume in one second; FVC, forced vital capacity

Figure 1 shows the relationship between BMI and pulmonary function at baseline FEV_1 _and FVC were positively correlated with BMI. However, a significant reduction in FEV_1_/FVC was noted in association with the higher BMI measures (*P *_trend _< 0.001 for both genders). During the 12 month follow up, as BMI rose FEV_1_/FVC lagged behind predicted values, generating a negative association between the change in BMI and the FEV_1_/FVC as a percent of predicted: -0.42 for boys (*p *< 0.001) and -0.44 for girls (*p *< 0.001) (Spearman rank correlation coefficient, r_s_). The average change in BMI during the follow-up was higher in male than females, 0.49 ± 1.24 and 0.24 ± 1.13 kg/m^2 ^(mean ± standard deviation), respectively.

An evaluation of the relationship between BMI and asthma risk factors, identified several gender specific statistical associations (Table 3). These included exercise frequency, parental education and pet ownership in boys, and breastfeeding in girls. As a result, two multivariate analyses of the relationship between BMI and asthma risk were conducted by gender. Model 1 (Table 4, upper panel) was adjusted for the significant covariates identified in the univariate analysis (Table 1) including breastfeeding, fungus/mould exposure, parental asthma, parental education, initial age, BMI (underweight, normal, overweight, and obese) and change in BMI (kg/m^2^) during the follow-up. Model 2 adjusted further to add in exercise frequency, air conditioning usage, cigarette smoking, ETS, and pet ownership (full model with all covariates, Table 4, lower panel). Despite the added covariates, the association between BMI and the risk of developing asthma remained significant for girls. Although the odds ratio for asthma risk increased progressively with increasing BMI in girls at baseline, the change in BMI at follow up was not a significant determinant of risk. Female adolescents who were overweight or obese to start were respectively at a 12% and 75%, greater risk of developing asthma than those with normal weight. This pattern, however, was not observed in boys. There was also no statistical association observed between BMI and asthma in the underweight category for either gender.

**Table 3:** The association between demographic variables and body mass index by gender at baseline

Demographic variables	BMI category				
		
	Underweight	Normal	Overweight	Obese	*p*-value *
**Boys (No. of subjects)**	307	1327	276	296	
Breastfeeding (%)					0.74
No	56.4	58.6	56.9	59.5	
≤ 1 month	20.5	17.1	15.9	16.2	
> 1 month	23.1	24.3	27.2	24.3	
Regular exercise (%)					< 0.001
Seldom	6.5	9.0	5.8	12.8	
Sometimes	40.7	35.7	40.6	46.3	
Regularly	52.8	55.3	53.6	40.9	
Air conditioning (yes, %)	87.6	89.0	89.9	93.9	0.06
Cigarette smoking (yes, %)	12.4	8.1	8.3	10.1	0.17
Exposure to ETS (yes, %)	53.8	56.0	55.8	63.5	0.07
Mould/Fungal at home (yes, %)	27.0	28.2	27.5	28.7	0.97
Parental history of asthma (yes, %)	2.3	3.5	2.2	3.7	0.41
Parent education (%)					0.03
≤ 9 yrs	41.7	32.5	29.7	35.1	
10 - 12 yrs	29.0	36.5	34.4	34.1	
≥ 13 yrs	29.3	31.1	35.9	30.7	
Pet (yes,%)	30.6	25.5	22.1	30.4	0.04

**Girls (No. of subjects)**	204	1201	251	190	
Breastfeeding (%)					0.04
No	52.0	57.9	55.0	48.4	
≤ 1 month	17.3	17.4	14.7	15.8	
> 1 month	30.4	24.7	30.3	35.8	
Regular exercise (%)					0.58
Seldom	19.6	19.1	15.5	19.0	
Sometimes	59.8	57.2	63.8	59.0	
Regularly	20.6	23.7	20.7	22.1	
Air conditioning (yes, %)	84.8	89.2	88.1	88.4	0.43
Cigarette smoking (yes, %)	2.0	2.4	3.2	2.6	0.84
Exposure to ETS (yes, %)	52.5	58.2	59.4	60.0	0.38
Mould/Fungal at home (yes, %)	30.4	32.5	26.7	31.6	0.32
Parental history of asthma (yes, %)	5.9	4.9	5.2	4.2	0.89
Parent education (%)					0.75
≤ 9 yrs	31.4	34.3	32.7	34.7	
10 - 12 yrs	37.3	35.8	40.2	39.0	
≥ 13 yrs	31.4	29.9	27.1	26.3	
Pet (yes,%)	27.0	27.8	31.5	34.7	0.20

BMI, body mass index; ETS, environmental tobacco smoking.

* Generalized Mantel-Haenszel Chi-Square test (Fisher's Exact test was used for Cigarette smoking due to small sample size).

**Table 4:** Multivariate-adjusted odds ratios for physician-diagnosed asthma

Baseline characteristics	Boys		Girls	
	
	OR (95% CI)	*p*-value	OR (95% CI)	*p*-value
Model 1 *				
BMI				
Underweight	0.77 (0.49-1.19)	0.25	0.81 (0.50-1.29)	0.38
Normal	Reference		Reference	
Overweight	1.02 (0.67-1.54)	0.93	1.11 (0.75-1.65)	0.60
Obese	1.02 (0.68-1.52)	0.92	1.75 (1.18-2.59)	0.01
Change in BMI (per unit of BMI increase in kg/m^2^) during the follow-up	1.02 (0.91-1.14)	0.74	1.01 (0.90-1.14)	0.83
-2 Log Likelihood	1481.53		1473.71	

**Model 2 **†				
BMI				
Underweight	0.76 (0.49-1.18)	0.22	0.79 (0.49-1.26)	0.28
Normal	Reference		Reference	
Overweight	1.02 (0.67-1.55)	0.93	1.12 (0.76-1.67)	0.57
Obese	1.04 (0.69-1.56)	0.86	1.75 (1.18-2.61)	0.01
Change in BMI (per unit of BMI increase in kg/m^2^) during the follow-up	1.02 (0.91-1.14)	0.73	1.01 (0.90-1.14)	0.82
-2 Log Likelihood	1480.04		1465.11	

BMI, body mass index.

* Adjusted for significant covariates identified in univariate analysis, namely, breastfeeding, fungus/mould at home, parental history of asthma, and parent education along with age.

† Further adjusted for exercise frequency, air condition usage, cigarette smoking, ETS, and pet ownership (full model with all covariates).

Although there was no statistical relationship between changes in BMI and asthma during the follow up, the risk of having physician-diagnosed asthma appeared 1-2% higher for each unit increase in BMI. The Hosmer and Lemeshow tests for both models were non-significant, indicating no clear lack of fit. Nevertheless, Model 1 was a parsimonious model capable of characterizing asthma risk, given that the results of the likelihood-ratio tests were not significant for both boys (χ^2 ^= 1.49, df = 6, *p *= 0.96) and girls (χ^2 ^= 8.60, df = 6, *p *= 0.20). Thus, it appears that breastfeeding, history of parental asthma and parental education are important risk factors (*p *< 0.05) for asthma in boys. In contrast, baseline BMI, parental asthma and fungal/mould exposure are critical for asthma development in girls.

## Discussion

The overall prevalence of asthma in the male and female subjects screened was 10.0% (51,225/513,061) and 7.0% (35,604/504,857) respectively. However, the relative number of cases recorded within this study subset at follow up was slightly higher: males, 10.9% and females, 14.1%. Females seemed to have higher incidence. Among adolescents with undiagnosed asthmatic symptoms at baseline, the risk of developing physician-diagnosed asthma one year later increased significantly for girls with higher starting BMI, but not so for boys. The association persisted even after adjustment for other asthma risk factors such as breastfeeding and parental asthma history. Adolescents with higher BMIs tended to have greater FVC and FEV_1 _but lower FEV_1_/FVC ratios for both genders. Similar results were observed in previous studies [23-25].

Although the effect was not significant, the incidence of asthma tended to track changes in BMI during the 12-month follow-up. During the same interval, pulmonary function increased with increasing BMI but the FEV_1_/FVC ratio for instance was lower than predicted for the new BMI. The analyses do not indicate whether obesity limits chest mechanics or airway conductance or whether impaired lung function restricts physical activity and thus, predisposes one to increased BMI. However, the obesity appears to precede at least the clinical expression of the asthma. The combination could represent a vicious downward cycle of positive feedback. Structural changes in the airway wall that limit airflow may represent key determinants in the consequent pathology of asthma [26,27]. Changes in body mass associated with age and declining health may explain in part disease associated declines in pulmonary function and thus, provide additional critical evidence of a causal relationship between obesity and asthma consistent with the conclusion of previous weight change studies [11,25].

Baseline obesity was associated with the subsequent development of physician-diagnosed asthma in this cohort of subjects with preliminary or "preasthmatic" respiratory symptoms. This temporal coupling suggests that a latent pathphysiological process was already in place in the obese adolescents at start of the study. Although the mechanisms are unclear, several hypotheses have been proposed to explain the relationship between asthma and obesity. These mechanisms include obesity mediated systemic inflammation, airway hypersensitivity, and physical factors that restrict chest mechanics and/or airway compliance. Research suggested that obesity could facilitate asthma through a persistent, low-grade inflammation mediated by multiple cytokines, chemokines, and acute-phase proteins, such as interleukin-6 [28]. Those with a higher BMI tend to develop altered airway mechanics, reduced tidal volume and airway hyperresponsiveness (AHR) [28,29]. Despite an absolute increase in FVC and FEV_1_, relative lung function decreased with increasing total body fat after adjustment for height and weight [30]. In obstructive diseases, such as asthma, FEV_1 _is often reduced disproportionately compared to FVC, resulting in reduced FEV_1_/FVC ratios [31]. These interactions may explain current and previous observations [24] that obese adolescents have reduced FEV_1_/FVC ratios [32]. Proportional declines in both FEV_1 _and FVC, in obese, non-asthmatics may preserve FEV_1_/FVC ratios within normal limits [33], suggesting obesity may specifically aggravate pulmonary dysfunction in in a susceptible cohort of those with asthma or destined to develop asthma.

The impact of higher BMI on asthma risk might be influenced by other factors such as hormonal status. The strong relationship between obesity and asthma in females, suggests a possible interaction with ovarian hormones. Shore and Johnston suggest this hormonal hypothesis is supported by several observations. 1) The impact of obesity on asthma is strongest in girls who undergo puberty early. 2) The severity of asthma among those with early menarche increases with BMI. 3) Obesity advances the onset of puberty in females presumably through an effect on steroid synthesis or dynamics. 4) Estrogen replacement in post postmenopausal woman is associated with an increase in the incidence of asthma. 5) leptin appears to aggravate the pathophysiology of asthmatic and obesity appears to exaggerate leptin production in women compared to men [11].

There appear to be aged related clusters of asthma concentrated in children ages 1-4 and again in adolescence. Boys are concentrated in the early group and girls more so in the latter group. Since boys and girls follow very different temporal growth patterns it is unclear whether the difference arises from the influence of growth meadiators or whether this is more evidence for gonadal hormonal participation [7,29]. The earlier maturation of lung function in females may contribute to a reduced capacity to adapt to increasing BMI compared to boys whose lung function continues to develop into their early twenties [34]. As a result, if growth is a cofactor, a similar influence of obesity might emerge in males later in adolescence. Thus, inconsistent findings regarding gender effects [35] might be attributed in part to differences in the age of the subject populations studied [29]. Finally the inconsistencies may also pertain to other demographic factors or potential risk factors for asthma, for example: higher parental education may result in more consistent child care between genders. The current analysis revealed a significant difference between boys and girls in several risk factors for asthma, including exercise frequency, cigarette smoking, mould/fungal exposure at home, parental asthma, and pet ownership. The observed association between asthma and very low BMI among boys needs to be explored further with a large, prospective study. Although this study reaffirms the association between BMI and the risk of asthma, the etiologic mechanisms of BMI-associated asthma susceptibility remain unclear and will require further investigation.

A major strength of this study is its prospective design that allowed evaluation of the magnitude and temporal relationship between BMI and asthma risk. The result is consistent with the comprehensive review [11] suggesting that obesity precedes asthma in children and adolescent and that gender may play a role. The unique contributions in the current study include: 1) the acute time dependency of the asthma risk especially for obese adolescent female and 2) the clear value of simple questionnaire-based screening to identify those at high risk who might benefit from aggressive obesity management.

However, some limitations should be noted. First, although the prevalence of physician-diagnosed asthma in the current cohort compared well with self-reported asthma 8.5% (4.2% to 13% aged 12-17 yrs) during the same period [22], the results may not truly represent the prevalence of asthma in the general adolescent population in Taiwan. The current study population was a selected subgroup of adolescents with existing asthma-like symptoms and thus, may not represent the larger national cohort or the actual asthma prevalence or incidence of new asthma cases. However, this study was designed to focus on high risk groups for developing asthma during adolescence and the possibility of selection bias is low. Second, twelve months of follow-up may not be sufficient to detect effects of changes in BMI on asthma risk and lung functions that require years to evolve [23]. Both asthma and obesity often begin in early life [36], thus the impact of incremental changes in BMI later during adolescence may be less significant. However, if the premise is correct that the adolescent-obesity-asthma relationship is indeed dependent on the duration and degree of obesity, the implications for early prevention of asthma are obvious. Furthermore, although exercise frequency had been used to characterize physical activity [37], the possibility of residual confounding effects cannot be excluded. For instance, the intensity and duration of different exercises (e.g., baseball and jogging) may play a critical role in the association between BMI and asthma risk. The validity and accuracy of using exercise frequency (e.g., seldom, sometimes and regularly) also need to be addressed. Finally, although major confounding factors for asthma were taken into account, unmeasured factors such as diet and genetic polymorphisms may contribute a residual confounding influence [38]. The strength of the relationship of BMI to asthma risk might be improved and clarified if data on these variables could be obtained and analysed.

## Conclusions

This study found that higher BMI (BMI > 25 kg/m^2^) increases the risk for physician-diagnosed asthma among female, but not male, adolescents with preasthmatic symptoms, aged 13-15 yrs. Obesity in children and adolescents has increasingly become a worldwide epidemic. While further research is required to understand the mechanisms connecting asthma and obesity, targeted approaches such as weight management and early prevention of obesity is clearly prudent because of the considerable benefits for children and adolescents. This is particularly important for those at high risk for asthma.

## Competing interests

The authors declare that they have no competing interests.

## Authors' contributions

WCH conceived and designed the study, participated in the data collection, performed the analysis and drafted the manuscript. YSL and MHL and LM helped the design of the study, statistical analysis and interpretation of data. HTH and JLC participated in the interpretation of data and drafting the manuscript. PCC and RSL conceived and supervised the conduct of the study, and helped to draft of the manuscript. All authors read and approved the final manuscript.

## Pre-publication history

The pre-publication history for this paper can be accessed here:

http://www.biomedcentral.com/1471-2458/11/542/prepub
